# Determining the Population Frequency of the *CFHR3/CFHR1* Deletion at 1q32

**DOI:** 10.1371/journal.pone.0060352

**Published:** 2013-04-16

**Authors:** Lucy V. Holmes, Lisa Strain, Scott J. Staniforth, Iain Moore, Kevin Marchbank, David Kavanagh, Judith A. Goodship, Heather J. Cordell, Timothy H. J. Goodship

**Affiliations:** 1 Institutes of Genetic and Cellular Medicine, Newcastle University, Newcastle upon Tyne, United Kingdom; 2 Northern Molecular Genetics Service, Newcastle upon Tyne Hospitals NHS Foundation Trust, Newcastle upon Tyne, United Kingdom; Mario Negri Institute for Pharmacological Research and Azienda Ospedaliera Ospedali Riuniti di Bergamo, Italy

## Abstract

In this study we have used multiplex ligation-dependent probe amplification (MLPA) to measure the copy number of *CFHR3* and *CFHR1* in DNA samples from 238 individuals from the UK and 439 individuals from the HGDP-CEPH Human Genome Diversity Cell Line Panel. We have then calculated the allele frequency and frequency of homozygosity for the copy number polymorphism represented by the *CFHR3/CFHR1* deletion. There was a highly significant difference between geographical locations in both the allele frequency (*X^2^* = 127.7, DF = 11, P-value = 4.97x10^-22^) and frequency of homozygosity (*X^2^* = 142.3, DF = 22, P-value = 1.33x10^-19^). The highest frequency for the deleted allele (54.7%) was seen in DNA samples from Nigeria and the lowest (0%) in samples from South America and Japan. The observed frequencies in conjunction with the known association of the deletion with AMD, SLE and IgA nephropathy is in keeping with differences in the prevalence of these diseases in African and European Americans. This emphasises the importance of identifying copy number polymorphism in disease.

## Introduction

Complement genes within the RCA (Regulators of Complement Activation) cluster at chromosome 1q32 are arranged in tandem within two groups [1]. In a centromeric 360 kb segment lie the genes for factor H (*CFH*) (OMIM 134370) and five factor H-related proteins – *CFHR1* (OMIM 134371), *CFHR2* (OMIM 600889), *CFHR3* (OMIM 605336), *CFHR4* (OMIM 605337) and *CFHR5* (OMIM 608593). Sequence analysis of this region shows evidence of segmental duplications (SDs) resulting in a high degree of sequence identity between *CFH* and the genes for the five factor H related proteins [2]–[4]. SDs such as those seen in the RCA cluster are frequently associated with genomic rearrangements [5]. These usually occur as a result of non-allelic homologous recombination (NAHR) between SDs but can also be a result of gene conversion and microhomology mediated end joining (MMEJ) [6]. Genomic disorders at this locus have affected *CFH* and the *CFHR*s in a number of ways. Deletions as a result of NAHR lead to the loss of *CFHR1*, *CFHR3* and *CFHR4*. Deletions within genes, occurring through both NAHR and MMEJ, result in the formation of hybrid genes (*CFH/CFHR1*, *CFHR1/CFH*, *CFH/CFHR3*, *CFHR3/CFHR1*) associated with diseases such as atypical haemolytic uraemic syndrome (aHUS) and membranoproliferative glomerulonephritis (MPGN) [7]–[9]. Complete deficiency of factor H related proteins 1 and 3 had been found to be occur in ∼4% of a European population in protein studies before DNA studies of the region [10]. This DNA copy number polymorphism (CNP) has been extensively characterised in health and disease. It has been shown that the deletion is associated with the presence of factor H autoantibodies in aHUS [11], [12], with an increased risk of SLE [13] and a decreased risk of age-related macular degeneration [14] and IgA nephropathy [15], [16]. That there might be differences in the population frequency of the *CFHR3/1* deletion was suggested from a study published in 2006 which showed that the prevalence of homozygous deletion in African populations was ∼16% [17]. Population difference in the deletion have been confirmed in subsequent studies [13], [18], [19]. In this study we have measured copy number of *CFHR3* and *CFHR1* with multiplex ligation-dependent probe amplification (MLPA) [20] in a range of populations derived from the HGDP-CEPH Human Genome Diversity Panel (http://www.cephb.fr/en/hgdp/diversity.php) [21].

## Methods

### Ethics statement

Use of anonymous human DNA samples in this study was approved by the Northern and Yorkshire Multi-Centre Research Ethics Committee.

*CFHR1* and *CFHR3* copy number was measured in DNA samples from 238 individuals from the UK and 439 individuals from the HGDP-CEPH panel. The UK samples comprised 70 DNA samples from the Health Protection Agency Culture Collections (http://www.hpacultures.org.uk/products/dna/hrcdna DNA), 10 samples obtained from local blood donors and 158 DNA samples from control individuals within the Wellcome Trust Case Control Consortium [22], [23]. The samples from the Health Protection Agency were originally obtained from a control population of randomly selected, non-related UK Caucasian blood donors. The full collection of samples with the HGDP-CEPH panel consists of 1051 individuals from 51 world populations (http://www.cephb.fr). We selected for analysis 439 samples from 17 different countries comprising 25 different populations (Table 1). We did not include populations for which data is either already available (for example European populations such as France) or where samples numbers were too small to be representative. There were still some populations with a small number of samples, including the sub-Saharan region of Africa. These were combined into 11 geographical locations (Table 2) for subsequent analysis. In each of these locations the number of samples was greater than 20. In total 133 samples from African populations were analysed, including 83 from sub-Saharan countries. *CFHR1* and *CFHR3* copy number was measured as described previously [24] using multiplex ligation-dependent probe amplification [20] (MLPA) using a kit from MRC Holland (www.mlpa.com) (SALSA MLPA kit P236-A1 ARMD) and in house probes.

**Table 1:** HGDP-CEPH samples used for measurement of *CFHR3* and *CFHR1* copy number.

Country	Number of samples analysed	Populations (n)
Algeria	29	Mzab (29)
Brazil	22	Surui (8)
		Karitiana (14)
Central African Republic	23	Biaka pygmy (23)
China	50	Han (44)
		Dai (6)
Colombia	7	Colombian (7)
Democratic Republic of Congo	13	Mbuti pygmy (13)
Italy	49	North Italy (13)
		Tuscan (8)
		Sardinian (28)
Japan	29	Japanese (29)
Kenya	11	Bantu (11)
Mexico	34	Pima (14)
		Maya (20)
Namibia	6	San (6)
Nigeria	21	Yoruba (21)
Pakistan	50	Hazara (21)
		Burusho (25)
		Pathan (4)
Russia	41	Adygei (16)
		Russian (25)
Senegal	22	Mandenka (22)
Siberia	24	Yakut (24)
South Africa	8	Bantu (8)
TOTAL	439	

**Table 2:** Allele frequencies and counts of the *CFHR3/CFHR1* deletion in UK and HGDP-CEPH populations.

Geographical location	Allele frequency of the *CFHR3/CFHR1* deletion (%)	Counts (del^+ +^/del^+ −^/del^− −^)	Hardy-Weinberg	Allele frequency of the *CFHR3/CFHR1* deletion in individual populations (%)	P value compared to the UK population	P value compared to all other populations combined
UK (n = 238)	18.3	8/71/159	χ^2^ = 0, p = 0.99			0.941
South America^1^ (n = 29)	0.0	0/0/29		Surui 0.0	3.53×10^−5^	1.24×10^−5^
				Karitiana 0.0		
				Colombian 0.0		
Japan (n = 29)	0.0	0/0/29		Japanese 0.0	3.53×10^−5^	1.24×10^−5^
Mexico (n = 34)	1.5	0/1/33	χ^2^ = 0.01, p = 0.99	Pima 0.0	6.75×10^−5^	2.90×10^−5^
				Maya 2.5		
Siberia (n = 24)	2.1	0/1/23	χ2 = 0.01, p = 0.99	Yakut 2.1	0.00189	9.97×10^−4^
China (n = 50)	6.0	0/6/44	χ2 = 0.2, p = 0.90	Han 6.8	0.0153	6.12×10^−4^
				Dai 0.0		
Pakistan (n = 50)	15.0	0/15/35	χ2 = 1.56, p = 0.46	Hazara 14.2	0.479	0.499
				Burusho 12.0		
				Pathan 37.5		
Italy (n = 49)	22.4	3/16/30	χ2 = 0.19, p = 0.91	North Italy 15.4	0.326	0.275
				Tuscan 18.7		
				Sardinian 26.8		
North Africa^2^ (n = 29)	22.4	2/9/18	χ2 = 0.34, p = 0.85	Mzab 22.4	0.476	0.384
Russia (n = 41)	25.6	3/15/23	χ2 = 0.06, p = 0.97	Adygei 41.2	0.131	0.0756
				Russian 14.0		
Sub-Saharan Africa^3^ (n = 83)	33.7	7/42/34	χ2 = 1.44, p = 0.49	Biaka pygmy 8.7	8.37×10^−5^	2.42×10^−7^
				Mbuti pygmy 38.5		
				Kenya Bantu 50.0		
				San 8.3		
				Mandenka 50.0		
				South African Bantu 43.8		
Nigeria (n = 21)	54.7	7/9/5	χ2 = 0.38, p = 0.83	Yoruba 54.7	5.85×10^−7^	5.63×10^−8^

^1^Brazil, Colombia; ^2^Algeria; ^3^Central African Republic, Democratic Republic of Congo, Kenya, Namibia, Senegal, South Africa.

The P value derived using Fisher's exact test compares the allele frequency of the *CFHR3/CFHR1* deletion in HGDP-CEPH populations to that of the UK population or all other populations combined.

### Statistics

Chi-square analysis was used to test whether there was deviation from Hardy-Weinberg equilibrium in the geographical locations. A p value of <0.05 was considered to be not consistent with Hardy-Weinberg equilibrium. Chi-square analysis was undertaken to determine whether there was a significant difference between geographical locations in either the allele frequency of the *CFHR3/CFHR1* deletion, the genotype frequencies (del^+ +^/del^+ −^/del^− −^) or the frequency (del^+ +^) of a homozygous deletion of *CFHR3/CFHR1*. Fisher's exact tests were undertaken to determine whether in different geographical locations either the allele frequency of the *CFHR3/CFHR1* deletion, the genotype frequencies (del^++^/del^+−^/del^−−^) or the frequency of a homozygous deletion of *CFHR3/CFHR1* were significantly different to the values for these variables in the UK population, or to their values in all other populations combined.

## Results

The allele frequency of the *CFHR3/CFHR1* deletion in the various geographical locations and the individual populations within these locations is shown in Table 2. There was no deviation from Hardy-Weinberg equilibrium in any of the geographical locations. The *CFHR3/CFHR1* deletion was not present in either the South American or Japanese locations. The highest allele frequency for the deletion was 54.7% in Nigeria. The deletion was present in all other locations studied, with allele frequencies of 1.5%, 2.1% and 6.0% in Mexico, Siberia and China respectively, 15% in Pakistan, 18.3% in the UK, 22.4% in Italy and North Africa, 25.6% in Russia and 33.7% in Sub-Saharan Africa. Differences in allele frequencies between locations were highly significant (*X^2^* = 127.7, DF = 11, P-value = 4.97×10^−22^). The level of statistical significance derived using Fisher's exact test for the allele frequency of the *CFHR3/CFHR1* deletion in geographical locations compared to the UK population is shown in Table 2. The allele frequency of the *CFHR3/CFHR1* deletion was significantly lower in South America, Japan, Mexico, Siberia and China; was not significantly different in Pakistan, Italy, North Africa and Russia but was significantly higher in sub-Saharan Africa and Nigeria.

The *CFHR3/CFHR1* deletion was not found in homozygosity in Mexico, South America, China, Japan, Pakistan or Siberia. The frequency of homozygous deletion was 3.4% in the UK, between 5–10% in Italy, Russia, North Africa and Sub-Saharan Africa, and 33.3% in Nigeria. Differences in genotype frequencies between geographical locations were highly significant (*X^2^* = 142.3, DF = 22, P-value = 1.33×10^−19^). Differences in the frequency of the homozygous del^++^ genotype were also highly significant (*X^2^* = 56.8, DF = 11, P-value = 3.66×10^−8^). The level of statistical significance derived using Fisher's exact test for the del^++^/del^+−^/del^−−^ genotype frequencies and for the frequency of the *CFHR3/CFHR1* deletion in homozygosity in individual populations, compared to either the UK population or all other populations combine , is shown in Table 3. The genotype frequencies were significantly lower in South America, Japan, Mexico, Siberia and China; were not significantly different in Pakistan, Italy, North Africa and Russia but were significantly higher in sub-Saharan Africa and Nigeria.

**Table 3:** Homozygous *CFHR3/CFHR1* deletion frequencies in UK and HGDP-CEPH worldwide populations.

Geographical location	Homozygous *CFHR3/CFHR1* deletion (%)	Homozygous *CFHR3/CFHR1* deletion (%) in individual populations	P value compared to the UK population (genotype frequencies)	P value compared to all other populations combined (genotype frequencies)	P value compared to the UK population(del^++^ frequencies)	P value compared to all other populations combined (del^++^ frequencies)
UK (n = 238)	3.4			0.415		0.434
South America (n = 29)	0.0	Surui 0.0	1.60×10^−4^	1.14×10^−4^	0.605	0.633
		Karitiana 0.0				
		Colombian 0.0				
Japan (n = 29)	0.0	Japanese 0.0	1.60×10^−4^	1.14×10^−4^	0.605	0.633
Mexico (n = 34)	0.0	Pima 0.0	4.63×10^−4^	2.97×10^−4^	0.601	0.392
		Maya 0.0				
Siberia (n = 24)	0.0	Yakut 0.0	0.00920	0.00906	0.999	0.619
China (n = 50)	0.0	Han 0.0	0.00938	0.00495	0.358	0.157
		Dai 0.0				
Pakistan (n = 50)	0.0	Hazara 0.0	0.578	0.298	0.358	0.157
		Burusho 0.0				
		Pathan 0.0				
Italy (n = 49)	6.1	North Italy 7.7	0.470	0.458	0.407	0.472
		Tuscan 0.0				
		Sardinian 7.1				
North Africa (n = 29)	6.9	Mzab 6.9	0.425	0.533	0.297	0.372
Russia (n = 41)	7.3	Adygei 17.7	0.219	0.148	0.209	0.418
		Russian 4.0				
Sub-Saharan Africa (n = 83)	8.4	Biaka pygmy 0.0	1.14×10^−4^	1.49×10^−7^	0.0720	0.080
		Mbuti pygmy 7.7				
		Kenya Bantu 18.2				
		San 0.0				
		Mandenka 13.6				
		South African Bantu 12.5				
Nigeria (n = 21)	33.3	Yoruba 33.3	2.06×10^−6^	3.41×10^−7^	3.50×10^−5^	1.22×10^−5^

The P value derived using Fisher's exact test compare either the genotype frequencies (del^++^/del^+−^/del^−−^) or the frequency of homozygous *CFHR3/CFHR1* deletion (del^++^) in HGDP-CEPH populations with that of the UK population or all other populations combined.

## Discussion

In this study we have used multiplex ligation-dependent probe amplification (MLPA) to determine the copy number of *CFHR3* and *CFHR1* in a variety of different geographical locations derived from the HGDP-CEPH collection. MLPA has the advantage over other techniques that have been used in that it provides a specific determination of copy number. We measured copy number of both *CFHR3* and *CFHR1* to determine the deleted allele frequency because measurement of *CFHR1* copy number alone is not specific to this allele as it also occurs with the *CFHR1/CFHR4* deletion [24], [25]. Using MLPA we have been able to determine both the allele frequency of the deletion and the frequency of a homozygous deletion. For statistical purposes we have set the UK population as our reference. The value of 3.4% for the frequency of a homozygous deletion in the UK population in this study is similar to values that we have obtained in previous studies [11], [24] (Table 4) and the allele frequency of the deletion is similar to that which we obtained on introduction of the MLPA assay (17.3% in Moore et al [24]). The latter value is similar to the frequency of 18.3% that we have obtained in this study.

**Table 4:** Table **4.** Reported population frequencies of the *CFHR3/CFHR1* deletion.

Population	Number of individuals	Allele frequency of *CFHR3/1 del*	*CFHR3/1* *del/del*	Method	Reference
UK	119	6.3%	1.6%	MLPA	[11]
UK	505	17.3%	3.0%	MLPA	[24]
France	70	8.6%	2.9%	MLPA	[48]
Spain	129	24%	7.0%	MLPA and WB	[25]
European American	275	19.8%	4.4%	MLPA	[13]
Asian	282	5.7%	0.7%	MLPA	[13]
Hispanic	196	17.8%	2.6%	MLPA	[13]
African American	106	42%	16%	MLPA	[13]
Austria	252		4.4%	WB	[10]
Germany	100		2.0%	WB	[11]
Tunisia	59		20%	WB	[18]
African American	347		15.9%	Gene specific PCR	[17]
Hispanic	266		6.8%	Gene specific PCR	[17]
European American	279		4.7%	Gene specific PCR	[17]
Chinese	94		2.2%	Gene specific PCR	[17]
HGDP African (sub-saharan)	127		17.3%	Gene specific PCR	[17]
HGDP North African	29		17.2%	Gene specific PCR	[17]
HGDP Middle Eastern	211		14.7%	Gene specific PCR	[17]
HapMap CEU	60	24.2%	8.3%		[19]
HapMap CHB	45	8.9%	0%		[19]
HapMap JPT	45	6.7%	0%		[19]
HapMap YRI	60	55%	28%		[19]
Coriell Diversity Panel African American	100	37%	17%		[19]
Coriell Diversity Panel Caucasian	100	21%	4%		[19]
Coriell Diversity Panel Chinese	100	4.5%	0%		[19]
Coriell Diversity Panel Mexican	100	13%	0%		[19]
HapMap III CEU	59	21.2%	1.7%		[19]
HapMap III TSI	90	24.4%	5.6%		[19]
HapMap III GIH	90	38.3%	18.9%		[19]
HapMap III MEX	55	11.8%	1.8%		[19]
HapMap III CHB	45	6.7%	0%		[19]
HapMap III CHD	50	3.5%	0%		[19]
HapMap III JPT	46	3.3%	0%		[19]
HapMap III ASW	53	34.0%	9.4%		[19]
HapMap III LWK	52	42.3%	19.2%		[19]
HapMap III MKK	149	23.8%	3.4%		[19]
HapMap III YRI	60	53%	27%		[19]

MLPA, multiplex ligation-dependent probe amplification.

WB, western blotting.

PCR, polymerase chain reaction.

CEU, Utah residents with Northern and Western European ancestry from the CEPH collection.

CHB, Han Chinese in Beijing, China.

CHD, Chinese in Metropolitan Denver, Colorado.

GIH, Gujarati Indians in Houston, Texas.

JPT, Japanese in Tokyo, Japan.

LWK, Luhya in Webuye, Kenya.

MEX, Mexican ancestry in Los Angeles, California.

MKK, Maasai in Kinyawa, Kenya.

TSI, Toscans in Italy.

YRI, Yoruba in Ibadan, Nigeria.

ASW, African ancestry in Southwest USA.

The values for the allele frequency of the deletion, the genotype frequencies, and the frequency of a homozygous deletion that we obtained for world-wide populations using the HGDP-CEPH collection show marked population differences with the highest frequencies being seen in African populations. The findings in the African groups are consistent with those reported (Hageman et al [17]) in African Americans and validate their findings in HGDP-CEPH African samples which were based on a gene specific PCR method that measured frequency of a homozygous deletion Subsequently there have been several other studies documenting the frequency of the *CFHR3/CFHR1* deletion in a range of populations. The results from these are shown in Table 4. The values in this study for both allele frequency and the frequency of homozygous deletion are consistent with previous studies particularly for the UK, Japanese, Chinese and Nigerian populations. We chose in this study to combine several populations from sub-saharan Africa as the numbers for each group were small. However, the study of Sivakumaran et al [19] suggests that for this region there are significant differences in the allele frequency of the deleted allele between tribes. For instance, they found an allele frequency of 23.8% in the Maasai tribe of Kenya compared to 42.3% in the Luhya. As can be seen in Table 2 we also observed differences in the allele frequencies of the different populations within this geographical location. For instance in the Biaka pygmyies the allele frequency was 8.7% compared to 50% in the Kenyan Bantus and Senegal Mandenka tribes. Recent studies documenting the genetic variation in this region show evidence of at least two different genetic groups derived from the North and South of the Kalahari [26], [27]. This may explain the differences in the allele frequency that we have seen in sub-saharan Africa. It is possible that ancestral African populations with a low allele frequency of the deletion were the ones which participated in the “out of Africa” dispersal with the associated bottleneck reinforcing the low allele frequency. That generally the current African populations with a low allele frequency of the deletion are Hunter-gatherers is compatible with this [28], [29]. The high allele frequency of the deletion in the African-American population is compatible with the allele frequency seen in the Yoruba and Mandenka [27], [30].

How in evolution has this deletion arisen and how can the population differences be explained? The alternative pathway of complement is thought to be the oldest component of the innate immune system [31]. The earliest components of the alternative complement pathway to have been recognised are activators such as C3 which has been identified in a coral [32] suggesting their presence in the Cnidria. Regulatory components have been first recognised in the Agnatha with for instance identification of a C3 cleaving short consensus repeat protein in lamprey [33]. A protein (called SBP1) with a high degree of homology to human factor H was first described in the teleost, sand bass [34], [35]. Factor H has also been identified in the zebrafish [36]. In the zebrafish, the mouse and humans there are genes encoding SCR proteins with a high degree of homology to factor H in close proximity to the gene encoding factor H. In man there are the five factor H related proteins (*CFHR1-5*), in the mouse there are three factor H related proteins and in the zebrafish there are 4 factor H like proteins. Sequence analysis of this region in man suggests that these genes have arisen through a series of segmental duplication events [2]. Analysis of primate genomes undertaken by Sivakumaran et al suggests that chimps have more extensive duplication in this region than humans. The analysis also suggests that the duplications arose in a common ancestor of the chimp and humans after divergence from the orang-utan [19]. The duplicated segments predispose to both non-allelic homologous recombination (NAHR) and gene conversion [37]. The available evidence would suggest that the *CFHR3/CFHR1* deletion has arisen through NAHR after the initial formation of the SDs. Sivakuram et al used phylogenetic and linkage equilibrium analysis to determine the ancestral orgin of the deletion [19] and found a single origin in Caucasians and Asians but a recurrent origin in Africans. We believe that in certain populations that the deletion has resulted in an evolutionary benefit. There is evidence to suggest that polymorphisms in complement proteins are associated with susceptibility to infection [38]. For instance mannose-binding lectin (MBL) binds to microbes and activates the lectin pathway. Allelic variants in the gene (*MBL2*) encoding this protein are associated with differences in both the serum level and function of MBL. The frequency of these allelic variants differs in populations; and the same variants are associated with a differential risk of pneumococcal disease and leprosy. Recently it has been shown that variants in *CFH* and *CFHR3* are associated with susceptibility to meningococcal disease [39]. These observations taken with the knowledge that complement plays a significant role in the pathogenesis other diseases such as malaria [40] would suggest that infection has driven the geographical variability seen in complement variants such as the *CFHR3/1* deletion.

Since the *CFHR3/CFHR1* deletion was first described a number of studies have documented strong linkage disequilibrium of the deletion with common *CFH* haplotypes [41], [42]. In some populations the deletion is present on haplotypes H1-5 and absent on H6-7 [41]. In other populations the H2 haplotype perfectly tags the deletion [15]. Likewise in some populations individual SNPs have been shown to be in complete LD with the deletion. Zhao et al found that the deletion was in complete LD with rs6677604 in European Americans but not in African Americans (r^2^ = 0.60). Whether the deletion confers an independent risk for AMD, SLE and IgA nephropathy or is simply associated with protective/at-risk haplotypes is an area of controversy [19], [41], [43]. However, factor H related protein 1 blocks the C5 convertase but binds, in competition with factor H, to host surfaces through its C-terminal regulatory domain [44]. We are, therefore, of the opinion that deletion of *CFHR1* has a dual effect with reduced inhibition of terminal complement pathway activity but increased regulation by factor H of the alternative pathway. This may also explain why in some diseases (AMD and IgA nephropathy) the deletion is protective whilst in other others (SLE) it is associated with increased risk.

It is also possible that *CFHR3* has functional activity that contributes to the disease association seen with the *CFHR3/1* deletion. In African Americans with a higher frequency of the deletion the prevalence of AMD and IgA nephropathy is lower than in European Americans [45], [46] whereas the prevalence of SLE is higher [47]. Thus studying the population frequency of disease associated CNPs such as the *CFHR3/CFHR1* deletion provides novel insights into the pathogenesis of such diseases. However, at an individual level we do not think that screening for the deletion in the normal population is currently of any clinical benefit.
